# RE: Communicative competence of generative artificial intelligence in responding to patient queries about colorectal cancer surgery

**DOI:** 10.1007/s00384-024-04677-w

**Published:** 2024-07-05

**Authors:** Hinpetch Daungsupawong, Viroj Wiwanitkit

**Affiliations:** 1 Private Academic Consultant, Phonhong, Lao People’s Democratic Republic; 2 Department of Research Analytics, Saveetha Dental College and Hospitals, Saveetha Institute of Medical and Technical Sciences, Saveetha University, Chennai, India

Dear Editor,

We would like to discuss material arising from this published article “Communicative competence of generative artificial intelligence in responding to patient queries about colorectal cancer surgery [1].” In summary, the study compared the responses from a colorectal cancer (CRC) information book to the response capabilities of generative artificial intelligence (GAI) tools when it came to answering questions concerning colorectal cancer surgery in Korean. The results showed that, for a number of evaluation criteria, GPT-4, Google Bard, and CLOVA X performed comparably to the CRC information book. The conclusions’ generalizability is called into question by a few constraints, including the CRC book’s perhaps out-of-date content and the small sample size of evaluators. Furthermore, the lack of a direct assessment of hallucinations in GAI responses in the study may have affected the veracity and correctness of the data presented.

Using a CRC information book from 2020, which might have featured out-of-date material, was one of the method’s shortcomings. This might have inflated the accuracy of GAI by distorting the comparison between the book and GAI responses. Furthermore, bias may have been introduced into the results due to the small number of assessors, particularly when they were divided into MDT and patient groups. Furthermore, the study did not specifically examine whether or not GAI responses included hallucinations, which is important to consider when assessing the accuracy of the data presented.

One possible query for the author would be whether they took into account the effect that out-of-date information in the CRC book would have on the comparison with GAI replies. It would also be helpful to know how the study took into consideration any biases that might have been caused by the limited sample size of assessors and the literary style differences between the GAI responses and the CRC book. Furthermore, the study skipped over what might be a crucial area for additional research: the possible effects of errors or hallucinations in GAI responses on patient decision-making and clinical results.

Uncovered topics in the global literature that require greater investigation include a thorough examination of how out-of-date data affects the comparison of GAI replies and the CRC book. Furthermore, it could be insightful to look into the long-term implications of using GAI for medical information in patient decision-making and clinical outcomes. Prospective avenues for investigation may comprise evaluating the efficacy of therapies aimed at mitigating hallucinations in GAI reactions and enhancing the precision and dependability of the data furnished to patients. Additionally, looking at how GAI functions in shared decision-making and patient education in healthcare settings may provide important insights into how it may be used to enhance patient outcomes.

## Data Availability

No datasets were generated or analysed during the current study.

## Declarations

### AI declaration

The author use language editing computational tool in preparation of the article.

## References

[1] Jo MH, Kim MJ, Oh HK, Choi MJ, Shin HR, Lee TG, Ahn HM, Kim DW, Kang SB (2024) Communicative competence of generative artificial intelligence in responding to patient queries about colorectal cancer surgery. Int J Colorectal Dis 39:94. 10.1007/s00384-024-04670-3 38902500 10.1007/s00384-024-04670-3 PMC11189990

